# BioDEAL: community generation of biological annotations

**DOI:** 10.1186/1472-6947-9-S1-S5

**Published:** 2009-11-03

**Authors:** Paul Breimyer, Nathan Green, Vinay Kumar, Nagiza F Samatova

**Affiliations:** 1 North Carolina State University, Raleigh, C 27695, USA; 2 Oak Ridge National Laboratory, Oak Ridge, TN 37831, USA

## Abstract

**Background:**

Publication databases in biomedicine (e.g., PubMed, MEDLINE) are growing rapidly in size every year, as are public databases of experimental biological data and annotations derived from the data. Publications often contain evidence that confirm or disprove annotations, such as putative protein functions, however, it is increasingly difficult for biologists to identify and process published evidence due to the volume of papers and the lack of a systematic approach to associate published evidence with experimental data and annotations. Natural Language Processing (NLP) tools can help address the growing divide by providing automatic high-throughput detection of simple terms in publication text. However, NLP tools are not mature enough to identify complex terms, relationships, or events.

**Results:**

In this paper we present and extend BioDEAL, a community evidence annotation system that introduces a feedback loop into the database-publication cycle to allow scientists to connect data-driven biological concepts to publications.

**Conclusion:**

BioDEAL may change the way biologists relate published evidence with experimental data. Instead of biologists or research groups searching and managing evidence independently, the community can collectively build and share this knowledge.

## Introduction

Over the past decade, systems biology research has undergone two key transformations. On the one hand, public databases of experimentally generated *-omics *data are increasing in number, size and diversity, along with annotations predicted from these data by computational tools. Such annotations may include the predicted protein functions as part of genome annotation pipelines, the predicted high resolution 3-dimensional structures of proteins from amino acid sequence information alone, the predicted protein-protein interactions and interaction networks derived from databases of yeast-2-hybrid, or mass spectrometry pull-down experiments.

On the other hand, the number of on-line research articles, many of which are open access, is continually growing. There are currently over 20 million scientific abstracts in MEDLINE, growing at 500,000 articles per year [1]. Such articles often report the discovered evidence (e.g., mutagenesis experiments) for various hypotheses derived via mining these heterogeneous databases of publicly available data and annotations. For example, GenBank [2] may report the predicted annotations for the two genes, designated omcB and omcC, to encode putative outer membrane polyheme c-type cytochromes, which are important for Fe(III) reduction by *Geobacter sulfurreducens *bacteria. Later, the open access paper by Prof. Lovley's lab (PMID 12644478) may report experimental evidence indicating that OmcB, but not OmcC, has a major role in electron transport to Fe(III) in this organism.

The interesting question is how to close the growing gap between these two paths of scientific discovery in biomedical sciences: the *data-to-annotations-to-databases *path and the *annotations-to-hypotheses-to-evidence-to-publications *path. Essentially, what is the proper infrastructure to enable streamlining published evidences, which rely on upstream database annotations, into the databases, thus establishing a feedback loop into the database-publication cycle? Without such an infrastructure, it is quite likely that highly valuable knowledge extracted by researchers, who browse the databases for valuable annotations and spend tedious efforts to support their findings with possibly published evidences, is recorded only in researchers' personal notes and is not integrated into the database-publication cycle to assist other researchers.

**Figure 1:**
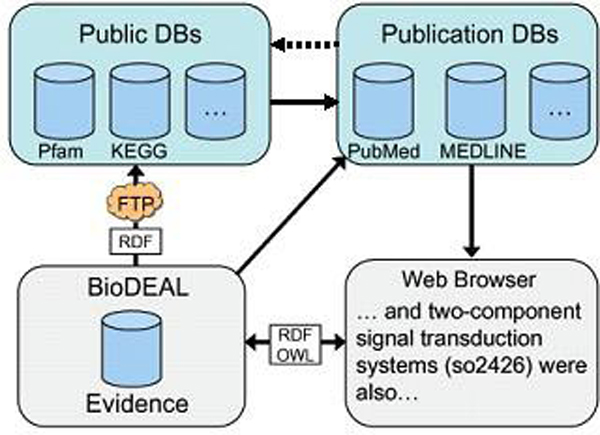
**The database, BioDEAL and publication feedback loop**.

In this paper we present and extend BioDEAL [3], a community *Bio*logical *D*ata-*E*vidence-*A*nnotation *L*inkage system that introduces a feedback loop into the database-publication cycle to allow scientists to make connections between data-driven biological concepts and publications, and vice versa. The cycle is illustrated in Figure 1. By subscribing to the services provided by BioDEAL, an end-user can annotate the facts reported in literature, associate them with semantic concepts, link them to semantically annotated biomedical events or relationships, and share these literature annotations with other researchers in a social network. For example, while reading the paper by Lovley et. al (PMID 1264447), the genome annotation expert may decide to link the omcB (GSU2887) gene with electron carrier activity (GO:0009055) in the Gene Ontology (GO) [4] and add a comment on experimental validation of its predicted function as the Fe(III)-reductase. Likewise, the reader interested in *Shewanella oneidensis *may annotate the event reported by Thompson et. al (PMID 11823232) that the chromate shock in *Shewanella *causes the repression of omcA and omcB genes and significant up-regulation of two-component signal transduction systems (SO_2426).

BioDEAL will record these annotations in a structured (XML) format so that other databases, such as GenBank or UniProt, may parse this information and potentially update its "Related Articles in PubMed" field for this gene page with the PubMed ID. Typically, public databases reference publications on the original genome sequencing and annotation. Other references are not captured, such as those associated with the genes, pathways or subsystems-level and the events or relationships between them, for example. It is typically very tedious work for people studying a particular system/gene(s) to track this information through time-consuming literature reading. Although a number of databases and frameworks can benefit from and/or enhance the functionality of BioDEAL, to the best of our knowledge, BioDEAL is the first system that enables such a feedback loop into the database-publication cycle.

## Methods

### Architecture

**Figure 2:**
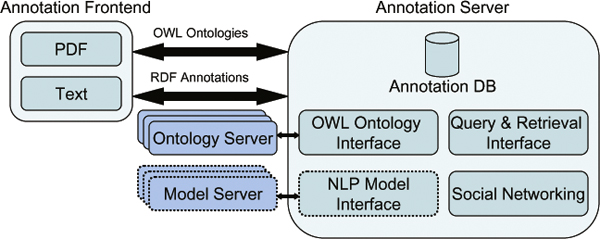
**The framework architecture - dotted lines indicate work-in-progress**.

**Figure 3:**
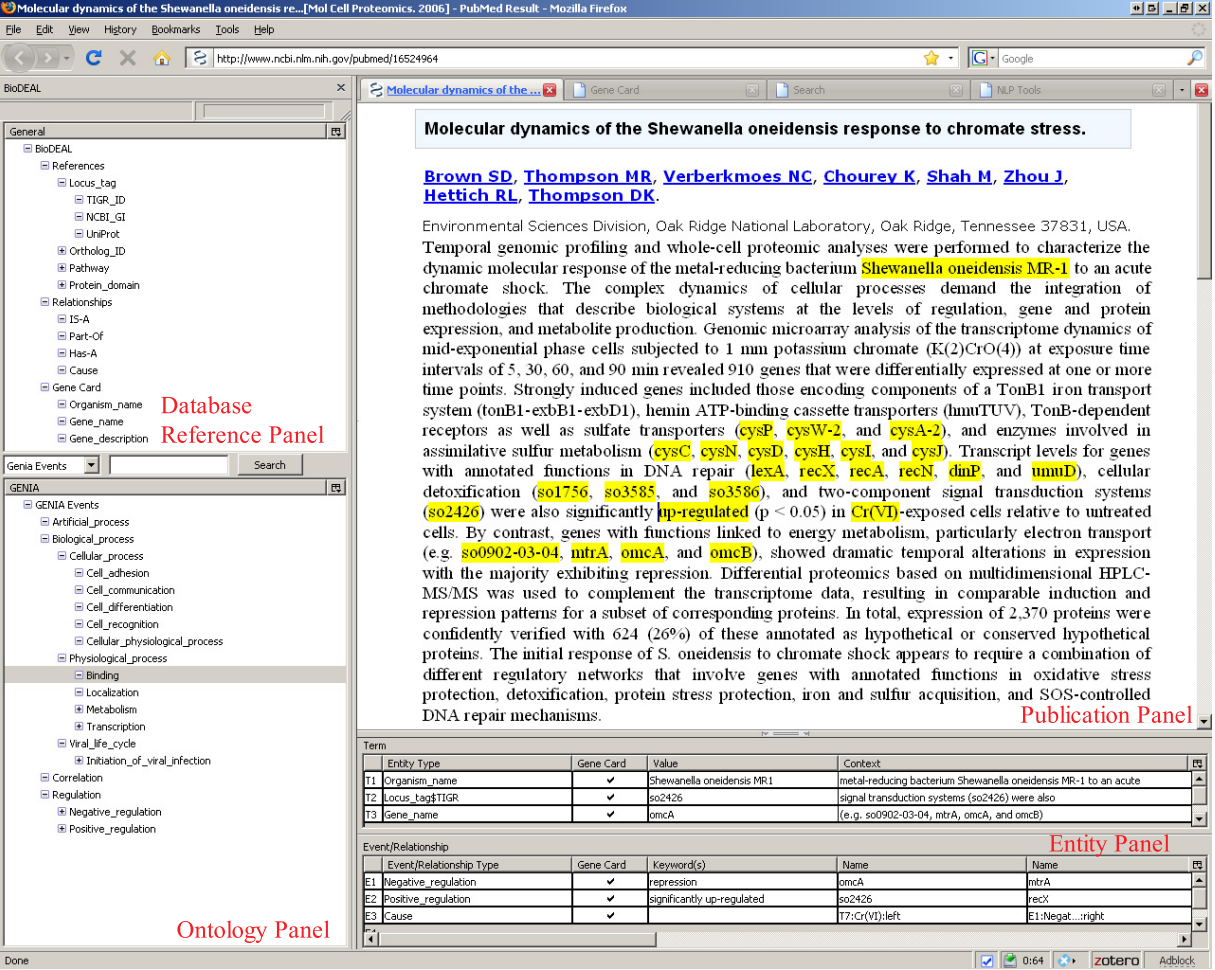
**The BioDEAL Browser Frontend**.

The current implementation of the BioDEAL framework consists of the following main components (Figure 2): an Annotation Server, the Annotation database, an OWL Ontology Interface, a Query and Retrieval Interface, a Social Networking block, and an Annotation Frontend. The Annotation Frontend, shown in Figure 3, is implemented as a web application to allow distributed users to annotate websites. The frontend is a Firefox plugin that supports two interfaces: one handles standard text and builds upon the W3C Annotea project and the other supports PDF documents. The user experience for both interfaces is similar: users highlight words or phrases to annotate and link them to semantic tags in the ontology tree by dragging or double-clicking the tree node for a highlighted phrase.

The BioDEAL framework lets users expose any ontology by extending a Java class or implementing specific web services. The OWL Ontology Interface sends available ontologies from the server to the Annotation Frontend in Web Ontology Language (OWL) format. Once an ontology is loaded the Annotation Frontend can query the OWL Ontology Interface for specific ontological categories via a search method. BioDEAL uses OWL for ontology communication because it is a W3C standard and will help enable others to develop new semantic tags and relationships as well as ease the development of new Annotation Frontends. The web services for incorporating ontologies and Natural Language Processing models, described below in the *Semi-Automation via NLP Tools *section, into BioDEAL uses REST technology, rather than the more common SOAP protocol, or XML-RPC. The REST protocol is straightforward and uses Uniform Resource Locator (URL) addresses to make calls. SOAP is a powerful protocol, but it introduces levels of complexity and redundancy that are not justified for our framework, and XML-RPC is also more complex than REST. The Annotation Server handles the communication between the Annotation Frontend and the backend BioDEAL database, which uses MySQL 5. Communication between clients and servers uses XML, and specifically either RDF or OWL, depending on the request context.

### Normalization of semantic concepts through ontologies

Ontologies are tagging formats that can support the semantic enrichment of data by embodying the abstract knowledge contained in the data, which can be used for data integration and analysis. Ontologies are used in BioDEAL to standardize the semantic concepts used for annotating entities, which allows data exchange between public databases and BioDEAL, and enables powerful user searches for evidence, which is displayed in gene cards.

Data tagging is especially useful in biological data due to the notorious complexity of the data formats and semantics. This is further compounded by the extremely rich and difficult vocabulary that is foreign to most non-biologists, necessitating that biologists play an active role in the interactive development process of the tagging. Furthermore, using standard tagging descriptions greatly increases the usefulness of tagged data because scientists in different research groups, backgrounds and locations can easily exchange and understand the enriched data.

BioDEAL allows users to incorporate any ontology into the system. Users may submit specific ontologies to the framework, such as the Systems Biology Ontology (SBO) and Gene Ontology (GO), both of which are expressed in OWL format. SBO and other ontologies can be found in The Open Biomedical Ontologies (OBO) Foundry [5]. BioDEAL backend clients receive OWL data by sending requests to the server, which directly queries the appropriate ontology and returns a subset of the data to the client. Using the server as an OWL gateway allows BioDEAL to manage the amount of data sent to the client.

The ontology data format will vary among different ontologies. Therefore, we are unable to design an ontology manager that uniformly communicates with all ontologies. Instead, we provide an OWL interface that must be implemented per ontology. While the communication uses OWL, the underlying ontologies may be in any format. The primary functionality of the interface is to return the immediate children of a particular node.

Users can submit ontologies to the framework by publishing a webservice or writing a Java class that extends the *Ontology *abstract class. Both approaches require two methods: *getRootNode *and *getOntologyNode*. The former returns the root node of the ontology, and the latter returns any child node. The object returned is an *OntologyNode*, which contains information about the current node and its children. The webservice returns the *OntologyNode *in serialized XML. We believe this interface is relatively straightforward to implement and will not impose an undue burden on ontology authors. All ontology communication between the BioDEAL server and client uses OWL, although non-OWL compliant ontologies can be published in the framework by following the same approach. Due to space limitations the OWL format is omitted.

BioDEAL further enriches ontologies by classifying each annotated word phrase as a term or relationship; the default is a term. Entities can also be grouped with a *Locus_tag*, which causes all marked entities to appear in the grouped gene's card, discussed below in the *Gene Cards *section.

### Tagging

Text Mining (TM) is a key technology for future bio-medical research [6]. Within Biological TM there are two forms of annotations: linguistic, such as part-of-speech (POS), and biological annotations that are created by biologists and aim to identify biological information in text. Linguistic annotation can leverage existing cross-domain annotation frameworks, whereas biological annotations are domain specific. Within biological annotations there are term annotations, which are un-analyzable basic units, and relationship, or event, annotations, which are more complicated and analyzable [7]. There are few current predictive tools that identify relationship events and their effectiveness is limited. Therefore, manual curation of this information by biologists is critical.

To create a relationship, users highlight an entity node, e.g., the protein *ahd*, and double-click the appropriate relationship node, e.g., *Binding*. The relationship will appear on the Entity panel as *relationship:value*, e.g., *Binding:ahd*. To associate other entities to the relationship, users highlight another phrase and either drag it onto the desired relationship value in the lower left panel or double-click the value. The panel will update to display the newly created link.

### High-throughput annotations

There are situations in which higher annotation throughput, compared to the manual approaches discussed so far, may be desirable for biologists. For example, the intrinsic scale of biological data often necessitates that biologists explore vast genomic sequences to answer questions pertaining to much smaller subgenomes. Coupled with page limit constraints imposed by publishers, biologists are often in possession of much more knowledge than is directly relevant or possible to include in publications. Rather than discarding this knowledge, authors may submit associated *supplemental *materials; in fact, some publishers strongly encourage them, such as *Molecular and Celular Proteomics (MCP)*. The supplemental documents often contain spreadsheets of biological facts that are associated with genes.

BioDEAL can leverage these documents to achieve higher annotation throughput by allowing users, either the publication authors or general readers, to upload supplemental spreadsheets to automatically annotate publications based on the biological information in the documents. Therefore, BioDEAL's approach is intended to be a natural extension of biologists' current practices.

BioDEAL users can submit supplemental spreadsheets for processing by clicking the *Browse *button in the *Upload *tab and navigating the desired comma separated value (CSV) file. The file will temporarily replace the Entity panel, and users can map ontological values to column names by dragging nodes from the Ontology or Database Reference panels to the column names, and the ontology values will appear in the second row of the table. When all desired columns are mapped, users can click the *Submit *button, causing BioDEAL to submit the file and mappings to the server to automatically create annotations. If one of the columns is mapped to *Locus_tag*, then BioDEAL will associate the annotations to the appropriate gene cards. The temporary file overlay table will be replaced by the Entity panel containing all annotations for the publication; the automatically generated annotations will be displayed in blue font to distinguish them from manual annotations.

**Figure 4:**
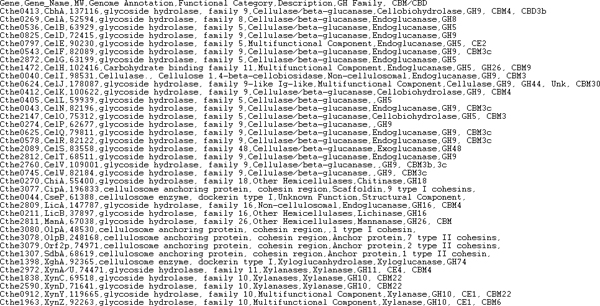
**An example annotation file in CSV format **[8]. The file may contain any number of columns, but must contain Gene and Gene name.

Due to the nature of supplemental materials, it is probable that some supplemental data will not appear explicitly in the publication. Rather than discard this information, BioDEAL creates annotations that are linked to the publication, thereby enabling search capabilities that can retrieve these relationships. BioDEAL also stores the uploaded files on the server and allows users to review them. Currently, BioDEAL supports automatic term annotation creation and future versions will support generating event annotations. An example CSV annotation file from the supplemental material for [8] is shown in Figure 4.

### Semi-automation via NLP tools

It is impossible for biologists to manually process all available data. Natural Language Processing (NLP) tools can provide semi-automatic semantic annotation of evidence such as human gene names by GIANT or protein-protein interactions by PIE (Protein Interaction Extraction) (see [9] for a survey of tools). GoPubMed automatically creates annotations through tools that align GO terms against PubMed abstracts [10] while Textpresso similarly uses regular expressions to match their custom ontology against full scientific texts [11]. Such annotations are essential for filtering data and providing the opportunity to inject supplemental tags into the data to support more robust and semantically meaningful analysis. Biologists can therefore focus their efforts on data that requires their attention, either because the model is unable to process it with a high degree of certainty, or to verify the integrity of the model and provide feedback for model calibration.

BioDEAL provides a Web browser tab to an NLP interface where users may execute NLP tools on publications to populate the BioDEAL database with automatically annotated evidence that is presented to the user through the User Frontend Interface (Figure 3). Model authors can submit NLP models to the framework in two ways: by creating webservices or extending an abstract Java class called *Model*. In both cases users must implement the *parseData(Object data) *method, which outputs an array of prediction objects that are inserted into the BioDEAL database, along with meta information regarding the tools and execution environment used. Semantic annotations from NLP tools are highlighted in green to indicate to the users that they are NLP-based predictions.

The web service approach involves wrapping the model in a light-weight web service interface that allows standard communication between BioDEAL and the model. The interface contains two components: a Predictor and a Refiner. The Predictor block implements tool-specific algorithms to automatically identify document entities. The Refiner block, which is optional, allows applications to analyze existing annotations to refine the behavior of the Predictor block.

While NLP tools can provide high-throughput and be effective at simple term identification, existing tools generally lack the maturity necessary to adequately process complex entity relationships because creating a semantically rich document representation is a hard problem. There is a lack of naming conventions, frequent abbreviations, common use of synonyms and homonyms, and terms often contain multiple words, such as: "human T-cell leukemia lymphotropci virus type 1 Tax protein" [12]. 53% of the corpus names in BioCreAtIvE, a community challenge to evaluate biological text mining, have more than one token [13]. Similarly, the acronym *ACE *has multiple meanings, including *angiotensin converting enzyme*, *afinity capillary electrophoresis*, *acetylcholinesterase*, although research has yielded correct acronym meaning detection with over 95% accuracy [14].

BioDEAL provides a mechanism to annotate these complexities, and the NLP tools may augment BioDEAL by automatically identifying simple terms in a document.

### Quality assurance and control

Annotation quality and quality control mechanisms are critical and non-trivial issues for a framework such as BioDEAL. Unlike manually annotated corpora by domain experts, annotations by web users will likely be noisy and inter-annotator discrepancies should be expected [7]. About 70 80% of annotations have inter-annotator agreement even if the annotators are instructed with well-formulated guidelines [15]. For example, the following sentence resulted in a GENIA annotation conflict: "*Calcineurin acts in synergy with PMA to inactivate I kappa B/MAD3, an inhibitor of NF-kappa B"*. One annotator identified a single event, which was "*Inactivation of I kappa B/MAD3 by Calcineurin." *However, another annotator claimed that the sentence conveys additional biologically important information: that calcineurin actually enables NF-kappa B to be activated by inactivating I kappa B/MAD, which inhibits NF-kappa B. For her, the expression *"I kappa B/MAD3, an inhibitor of NF-kappa B" *indicated another event: *"Inhibition of NF-kappa B by I kappa B/MAD3."*

Although BioDEAL does not automatically address such discrepancies, it can show all available annotations for a given term and let users decide how to resolve inconsistencies, either through re-annotation or augmenting the proper comments with the displayed annotations.

BioDEAL enabled annotated and curated web corpora can be utilized for manual curation by developers of public databases or NLP tools. It would be desirable for a framework, such as BioDEAL, to provide analytical intelligence to make decisions about collating and resolving possibly conflicting and uncertain annotations from potentially many users and/or various NLP tools. This is an open area of research, and deserves an active investigation.

Another source of errors comes from allowing the values for *Locus_tags *to be manually edited. Ideally, the terms should be semantically annotated only if they are present in the document. However, certain terms can be implicitly inferred from other terms, and are often not mentioned in the publication. For example, given a gene name (e.g., omcB), an organism name (*Geobacter sulfurreducens*) may be sufficient to infer the implied TIGR locus tag (e.g., GSU_2737) and is often omitted from the publication. As a result, the semantically annotated term (omcB) may be insufficient to automatically populate experimental evidence associated with this gene into public databases because a unique identifier is required, such as the locus tag. For this purpose, BioDEAL allows users to manually enter a value for the *Locus_tag *terms to facilitate the desirable streamlining of evidence recorded in publications directly to public databases that require these unique identifiers, although it inevitably raises data integrity concerns.

Public databases may use different *Locus_tags *for the same gene, and therefore the gene card specifies what type of *Locus_tag *is referenced (e.g., TIGR, UniProt). At this time, BioDEAL does not cross-reference *Locus_tags *between databases; instead, users must specify the appropriate database reference. In the future, users should be able to select the *Locus_tag *value from the BioDEAL interface.

Finally, determining publication IDs (e.g., PubMed ID) is potentially an error-prone process. It can be difficult because the BioDEAL plugin only has access to the source URL when a user opens a publication, which often does not include the publication ID. Instead, we created a publication ID lookup prototype that queries publication databases (currently only PubMed) with word phrases from a publication. Our initial findings are very encouraging: with only a few phrases of more than a few words we can identify the source publication with high certainty. As the number of annotated word phrases increases, so does our certainty. We will quantify and verify this observation when more user annotations are available. Likewise, an interface to prompt users to manually provide the PubMed ID or MEDLINE ID can be augmented with this approach.

## Results

### User frontend interface

BioDEAL supports a Web browser plugin interface that allows users to create and store document evidence while reading publications. The frontend contains the following core component panels: the Publication panel, the Database Reference panel, the Entity panel that contains the Term and Event/Relationship tables, and the Ontology panel, all shown in Figure 3. The Publication panel (top-right) contains the publication text, for example, from PubMed or MEDLINE; BioDEAL supports both PDF and text (HTML, PHP, etc.) documents. The former is typically a full publication identified by its URL on the journal web site, while the latter may be an abstract from PubMed.

The BioDEAL Database Reference panel (top-left) contains database indexable fields at various levels: gene level (e.g., TIGR or UniProt locus tag), protein domain level (Pfam or InterPro ID), or pathway level (KEGG or BioCyc ID). Such fields enable data exchange between public databases. It also contains common fields at the organism taxa level (e.g., organism name) and gene attributes level (e.g., function description). Although those fields do not typically have associated ID numbers, they are frequently used. For a similar purpose, this panel also includes event/relationship fields such as primitive associations (e.g., Is-A, Part-Of) as well as common interest ones (e.g., Cause, Binding, Up-regulation, Down-regulation, Expression).

The BioDEAL Entity panel (bottom) supports two entity types: terms and events/relationships (see the *Semantic Annotation of Terms *and *Semantic Annotation of Events and Relationships *sections). It contains a tabular list of terms and events annotated for a given publication. Each term annotation associates a word phrase in the publication with a semantic concept from the Ontology or Database Reference panels. For example, the word phrases *omcB*, *GSU2887*, and *Geobacter sulfurreducens *from the PMID:1264447 publication can be linked to the semantic concepts of *Gene_name*, *Locus_tag *and *Organism_name*, respectively, from the Database Reference panel. *GSU2887 *can be associated via a *HAS A *relationship with the 'electron carrier activity (GO:0009055)' GO node selected from the Ontology panel. Likewise, the 'outer membrane' phrase can be associated with the 'Localization' event concept in the GENIA ontology [7]. The BioDEAL Ontology panel (bottom-left) includes multiple ontologies such as GO and GENIA and supports extensions of other ontologies of interest, discussed in the *Normalization of Semantic Concepts Through Ontologies *section.

### Semantic annotation of terms

Semantic annotation of biomedical *terms *in a publication is an association of term semantic concepts (or keys) such as proteins, compounds, amino-acids, etc. with the word phrases (or values) in the publication, such as OmcB. The annotation process is quite simple; it involves highlighting a word phrase of interest in the Publication panel and double-clicking on the target semantic concept in the Database Reference panel or the Ontology panel. Similarly, a user may double-click on the target concept, which will create a new row in the Term table of the Entity panel and then drag the target term into the Value column in the table. Users can annotate terms in a document and link them with any number of semantic concepts. Each annotation action creates a row in the Term table with the (key, value) pair along with the context in the publication in which the term appears, which is displayed in the *Context *column. While only a few neighboring words around the term are shown in this column, mousing over the table cell will display the entire field. If a term contains multiple words that need to be jointly associated with the same term semantic concept, then BioDEAL assumes that these words appear as a continuous span in the text, which is supported by the previous study [16] concluding that 98% of terms appear in continuous spans.

Although terms denote semantically simple concepts, various ambiguities may arise depending on users and biomedical domains. To reduce such ambiguity, BioDEAL allows users to create key/value mappings with keys selected from the standardized semantic concepts that are created and agreed upon by user groups or domain experts; these normalized semantic concepts are called ontologies and are accessible through the Ontology panel (*Normalization of Semantic Concepts Through Ontologies *section). However, to accommodate domain-dependent definitions, for which standardized ontologies may not exist, BioDEAL provides ways to extend the supported ontologies. Mapping terms (e.g., omcB) to standard semantic concepts (e.g., gene_name), allows users and systems to conduct a dialogue with the same agreed upon terminology and enrich the types of searches that databases may choose to support. BioDEAL facilitates semantic associations that span multiple ontologies that may be concurrently used.

### Semantic annotation of events and relationships

BioDEAL also supports semantic annotation of biomedical events and relationships associated with semantically annotated biomedical terms. Examples may include the annotation of the Binding event through the linkage of a substrate term (e.g., malate) with a protein term (e.g., Mdh) or the annotation of a more complex Cause event through the linkage of the stress term (e.g., chromate shock) with the Up-regulation and Down-regulation events, which are, in-turn, linked with the proper gene-related terms (e.g., SO_2426 and omcB). BioDEAL supports semantic annotation of events expressed in terms of their related entities. An entity may be a term or another event. Therefore, BioDEAL supports *complex events *by allowing events to contain other events.

Users can create a semantic annotation of an event by double-clicking on the semantic concept denoting the event of interest from the Ontology or Database Reference panels. This action will create a new row in the Event/Relationship table in the Entity panel (see Figure 3). He/she may then drag-and-drop into that row previously annotated term and event rows from the Term and/or Event/Relation tables, respectively. The relationships may be unary, binary or k-nary, all of which are supported by BioDEAL. In addition, if the event directionality matters, BioDEAL allows each entity associated with the event to be labeled as either on the *Left *(an input) or the *Right *(an output). If an entity is bidirectional, then it appears as both *Left *and *Right*. The event ontology contains an attribute that specifies whether the events are directional (see the *Normalization of Semantic Concepts Through Ontologies *section).

Unlike terms, events are often not situated near related events or terms, so there is no corresponding *context *column in the Event table. Instead, the *Keyword *column serves a similar function for events by allowing users to highlight and drag keywords to the appropriate Keyword cell in the table. The *Event *table also contains an index column, the type of event (e.g., Up-regulation, Binding), an auto-generated *value *column constructed from the underlying terms, and a dynamic number of associated entities, which may be terms or other events.

### Gene cards

Many public databases (e.g., TIGR, UniProt, KEGG) support site-specific representations of fundamental genomic information called *gene cards*. Gene cards provide users with a simple interface to retrieve basic gene information in one place. Many of these databases present common fields, such as *Locus_tag *and *Gene_name*, and database-specific fields, such as *KEGG pathways*, as well as cross-references between fields from different databases, such as TIGR, NCBI-GI and UniProt locus tags. Existing gene cards, however, rarely contain records of relevant publications, and generally only include the original genome sequencing and annotation publications but not future evidence associated with specific genes, pathways, subsystems, etc. They also do not contain biological events, such as expression, binding and regulation, which could provide users with a new level of information.

**Figure 5:**
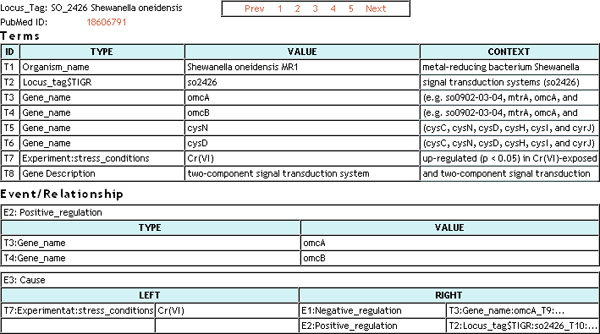
**A gene card for SO 2426 gene in Shewanella oneidensis MR-1**.

BioDEAL supports gene cards that organize all user annotated evidence regarding a gene by the source publications. To create gene cards, BioDEAL allows users to group entities from the entity tables related to the target gene of interest by selecting the check-boxes in the *Gene Card *column. The grouping must contain one Locus_tag row associated with the target gene. An example gene card derived from such a grouping is shown in Figure 5.

The underlying gene card representation is in XML/OWL, described in the *Data Accessibility *section, and can therefore be consumed by external database systems by linking to the fields from the Database Reference panel discussed in the *User Frontend Interface *section. All gene card information is present in the containing publication with the possible exception of the *Locus_tag *field, which is user-editable in case it does not appear in a publication (see the *Quality Assurance and Control *section).

Although the current BioDEAL implementation only supports gene cards, the system is extensible to other types and searches, such as Pathway cards or Gene Mutant cards, which we will investigate in future BioDEAL versions.

### Search

**Figure 6:**
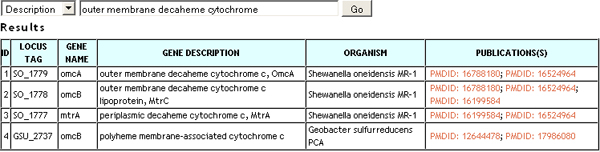
**Search page results showing different genes and the publication IDs**.

Users may access the BioDEAL gene cards through the BioDEAL search page. BioDEAL supports a simple query engine, shown in Figure 6, that allows users to search for data using some basic fields: PubMedID, *Locus_tag*, *Gene description*, *Gene_name*, etc. Each row in the search results corresponds to a gene card indexed by *Locus_tag*, along with gene-related information and the publication source (e.g., PubMed ID and hyperlink) in which the evidence recorded was reported. The *Locus_tag *is a hyperlink to the BioDEAL gene card. Basic gene-related information presented to the user includes, if available, the organism name for that gene, the common gene name, and the gene function description.

The search feature is available at the server web page. The request returns all evidence in the database that matches the search criteria, including any gene ID fields (e.g., Pfam, GenBank) and relationships. The search engine does not attempt to analyze the results prior to displaying them; if there are inter-annotator conflicts, all results are shown.

### Social networking

The BioDEAL server also contains a web portal with several features, including a social networking component where users can login, publish, and edit annotations within their permissions' sandbox. Collaborative features are becoming more crucial for scientists as researchers are increasingly geographically dispersed. Therefore, users can annotate as an individual or within communities. A scientist may publish annotations with different intentions: he/she may want to inform other scientists about a result, solicit opinions, or foster dialogue, to name a few. Our environment supports collaborative functionality, shown in Figure 2, by displaying annotations to users within their permission groups. By incorporating social networking features into BioDEAL, we hope to foster dialogue in the research community, and support a checks-and-balances system to improve the integrity of ontological annotations by allowing research group members to review the annotations of other members and make suggestions. While ontologies have been enthusiastically received in Biology, biologists may lack the background required to use them appropriately, and integrating social networking may produce better overall annotations. Annotations are public by default, but BioDEAL allows research groups to configure privacy settings that prevent non-group members from accessing their annotations. This feature can allow biologists to annotate evidence without exposing research-in-progress. In this scenario, the evidence is useful for the research group, but does not affect the data-publication cycle because the data cannot be consumed by public databases, unless the research group decides to make the evidence public at a later date.

### Data accessibility

The BioDEAL design supports storing information and recalling it in formats consumable by public databases, such as GenBank or UniProt. BioDEAL publishes OWL evidence files regularly on an FTP server that allows any system to retrieve public evidence. The primary keys in the evidence files are the BioDEAL Database Reference fields discussed in the *Results *section, with the *Locus_tag *field serving as the database cross-reference purpose. The success of our system as a feedback mechanism depends on whether existing systems incorporate BioDEAL into their life cycles.

During the course of annotating publication evidence, some data may not be attributed to a BioDEAL Database Reference field, and therefore will not be included in the FTP file. However, this data is still important because users can search on these fields. For example, consider a compound *X *that is not associated with a single gene. If evidence exists in BioDEAL for *X*, then users can query for all publications that refer to *X*.

## Discussion

The actual impact of web user annotated corpora creation remains to be seen, since BioDEAL is still a research tool, however, the potential benefits of such a framework are manifold. For example, public databases (e.g., TIGR, GenBank, BioDAS servers) can link to supporting evidence with predicted annotations. This may ultimately improve annotations through resolving inconsistencies and confirming their validity from published evidence.

Public databases can leverage BioDEAL annotation-evidence data using the FTP output files either directly or through an interface such as BioDAS [17], a communication protocol to exchange biological data annotations. Likewise, BioDEAL can present annotations generated by external projects such as BioCreAtIvE [9,18], whose overarching goal is to enhance abstracts with annotations. BioCreAtIvE users can gain from BioDEAL by querying our system to determine what has been annotated. BioCreAtIvE is limited to NLP tasks, which does not include many common tasks, such as identifying up and down regulation, therefore BioDEAL can augment BioCreAtIvE results.

Developers of NLP tools can generate annotated corpora beyond those currently supported (e.g., protein/gene names, functional and interaction annotations). NLP designers often lack the corpora necessary to develop their algorithms and BioDEAL builds corpora during its normal lifecycle, which NLP designers can use to reduce the difficulty of corpora generation, while improving the quality because they are manually curated by domain experts. We do not expect users to annotate every entity in a publication, however, it is likely that the key facts and evidence will be identified. Therefore, BioDEAL may be more suitable for the enrichment of public databases with evidence information, although the NLP community can likely benefit from this expert knowledge recorded through structured annotations.

Experts in different fields of biomedicine may exchange their annotations, comments, and open issues, thus improving the quality of genome annotations through collaborative knowledge creation. BioDEAL can help identify ambiguous annotations and facilitate community discussion and consensus on annotations, perhaps using an alert system when conflicting annotations are found. BioDEAL can also enable better classification of biological information and structured representation, while facilitating better search and information retrieval functionality beyond the standard available fields (e.g., author, affiliation, etc.).

## Conclusion

In this paper we presented the BioDEAL biological evidence and curation system that introduces a feedback loop into the database-publication cycle by allowing scientists to link experimental data-driven biological concepts to published evidence. BioDEAL supports a Web browser frontend that allows biologists to semantically annotate evidence from publications within a native browser setting. Evidence can be simple terms or complex events, and users can run NLP tools to facilitate semi-automation. External databases can use the reference fields (e.g., TIGR ID, Pfam ID) in the FTP output files to map evidence to their internal data. The social networking component allows the Biology community to annotate and curate evidence en masse, and provides a mechanism for research groups to verify the integrity of annotated evidence while providing a secure environment to conduct research.

Users can link evidence to specific genes and BioDEAL provides a *Gene Card *browser interface that allows users to search and review curated evidence. Similarly, users may use the BioDEAL search page to identify publications that contain user specified words, gene names, etc. BioDEAL also outputs evidence to OWL files that are available via FTP to allow other systems to consume the curated evidence. External databases can use the reference fields (e.g., TIGR ID, Pfam ID) in the FTP files to map evidence to their internal data.

BioDEAL may change the way biologists relate published evidence with experimental data. Instead of biologists or research groups searching and managing evidence independently, the community can collectively build and share this knowledge.

## Competing interests

The authors declare that they have no competing interests.

## Authors' contributions

All authors participated in the design and development of the framework components. PB primarily focused on the backend design and composing the manuscript; NG concentrated on the front-end design; VK focused on the PDF functionality and Social Networking design; and NFS formulated the overall problem, provided the vision for the BioDEAL design, oversaw the project development, and co-wrote the manuscript.
